# ﻿*Primulinapingnanensis*, a new species of Gesneriaceae from Guangxi, China

**DOI:** 10.3897/phytokeys.229.103735

**Published:** 2023-07-27

**Authors:** Zheng-Long Li, Yan-Yun Kuang, Qing-Qing Xu, Wei-Chuen Chou, Xin Hong, Li Ding

**Affiliations:** 1 Anhui Provincial Engineering Laboratory of Wetland Ecosystem Protection and Restoration, School of Resources and Environmental Engineering, Anhui University, CN-230601, Hefei City, Anhui Province, China Gesneriad Conservation Center of China Guilin China; 2 Gesneriad Conservation Center of China (GCCC), Guilin Botanical Garden, CAS, Guilin 541006, China Anhui University Hefei China; 3 Yunnan Key Laboratory for Integrative Conservation of Plant Species with Extremely Small Populations, Kunming Institute of Botany, Chinese Academy of Sciences, CN-650201 Kunming, Yunnan, China Kunming Institute of Botany, Chinese Academy of Sciences Kunming China; 4 State Key Laboratory of Soil and Sustainable Agriculture, Institute of Soil Science, Chinese Academy of Sciences, CN-210008, Nanjing, China Institute of Soil Science, Chinese Academy of Sciences Nanjing China; 5 Gesneriad Committee of China Wild Plant Conservation Association, National Gesneriaceae Germplasm Resources Bank of GXIB, CN-541006 Guilin, Guangxi, China National Gesneriaceae Germplasm Resources Bank of GXIB Guilin China; 6 Guangxi Key Laboratory of Plant Conservation and Restoration Ecology in Karst Terrain, Guangxi Institute of Botany, Guangxi Zhuang Autonomous Region and Chinese Academy of Sciences, CN-541006, Guilin City, Guangxi Zhuang Autonomous Region, China Guangxi Institute of Botany Guilin China

**Keywords:** Flora of Guangxi, Gesneriaceae, limestone flora, new taxon, *Primulinaorthandra*, taxonomy

## Abstract

A new species of *Primulina*, *P.pingnanensis*, from the Guangxi Zhuangzu Autonomous Region, China, is described and illustrated here. It is morphologically similar to *P.orthandra* but has significant differences in the bracts, corolla tube and lobes shape, as well as in the indumentum of the outer surface of the corolla, the filaments, the staminodes and the anthers. Colorful photographs and essential information of this new taxon are also provided, including detailed taxonomic description, distribution, habitat, the comparison table, and the IUCN conservation status. We also discuss a validation of new combination *P.crassifolia* and *Chiritacrassifolia*.

## ﻿Introduction

As of December 2022, about 250 taxa (including varieties) of *Primulina* Hance had been published worldwide, with 215 species and 18 varieties distributed in China and 18 species in Vietnam (GRC 2022; Wei et al. 2022). Based on the quantity and distribution of the *Primulina* species, it is apparent that the Karst limestone areas in Guangxi, China, are the center of *Primulina* diversity and distribution (Hao et al. 2014; Kong et al. 2017; Wei 2018; Li et al. 2019; Xu et al. 2019). This further indicates that more undescribed *Primulina* species may still be found in the Karst ecosystem of South and Southwest China (Möller 2019), especially in karst caves (Xin et al. 2018; Fu et al. 2022), cliffs and gorges.

Averyanov et al. (2020) described a new species of *Chirita* in North Vietnam, which they named *Chiritacrassifolia* Aver. & K.S.Nguyen. In 2022, after verification, Möller and collaborators transferred *C.crassifolia* to *Primulina* as *P.crassifolia* (Anh et al. 2022). *Primulinacrassifolia* (Aver. & K.S.Nguyen) T.T.P.Anh, F. Wen & Mich.Möller, comb. nov. Basionym: *Chiritacrassifolia* Aver. & K.S.Nguyen, Pl. Diversity Fl. Veg. Bat Dai Son 254 (2021). We emphatically revise the Latin name from ‘*Chiritacrassifolia*’ to ‘*Primulinacrassifolia*’ here and provide appropriate notes.

In March 2018, when we were on an expedition in Pingnan County, Guangxi, China, we found an unknown *Primulina*-like plant growing at the crevices of rocks on the top of a limestone cliff. According to its leaf arrangement (rosette), fleshy leaves, tubular corolla, and cracked fruits (capsule with parietal placentation), it should be included in *Primulina*. However, it did not match any known species of this genus. In addition, this species distinguishes from the genus *Petrocodon* by its lingulate stigma, curved filaments, and fleshy leaves. We collected specimens in the field and introduced some living plants into cultivation at the nursery of the National Gesneriaceae Germplasm Resources Bank of GXIB and the Gesneriad Conservation Center of China. The morphology of the cultivated plants has not changed from that of the wild plants during the past three years. We compared the collected specimens and living plants with other known type specimens and living plants of *Primulina*, and we found that it is distinctly different from other species of *Primulina*, especially those distributed in Guangxi and recently published species of *Primulina* (e.g., Du et al. 2021; 2022; Xiong et al. 2022; Pan et al. 2022), which are also different from this new taxon. We then confirmed that it was a new species.

## ﻿Material and method

The description is based on specimens collected in the wild. The measurements and morphological characteristics of the new species were taken from the type specimens processed by the authors. The type specimens of the new species were deposited in IBK. We examined *Primulina* specimens in IBSC, K, IBK, KUN, US, PE, and VNMN. We also searched two online herbaria, Chinese Virtual Herbarium (CVH, http://www.cvh.ac.cn) and Chinese Field Herbarium (CFH, http://www.cfh.ac.cn), to find species with similar morphology. We determined it to be an undescribed taxon. All morphological characters were studied under a dissecting microscope and were described using the terminology used by Wang et al. (1990, 1998).

## ﻿Taxonomic treatment

### ﻿ Primulina pingnanensis

Taxon classificationPlantaeLamialesGesneriaceae

Xin Hong, Z.L.Li & W.C.Chou. sp.nov.

2F9BA12D-CDE7-55E8-85FD-AAF6BC5A8F29

urn:lsid:ipni.org:names:77324168-1

Fig. 1

#### Diagnosis.

*Primulinapingnanensis* morphologically resembles *P.orthandra* but is distinguished from the latter by bracts lanceolate (*vs.* ovate), corolla tube funnel-form and no constriction in the middle (*vs.* tube near tubular with constriction in the middle), outer corolla surface sparsely white puberulent (*vs.* glabrous), corolla lobes oblong (*vs.* broadly ovate), filaments strongly curved at the middle (*vs.* straight), anthers fused from the entire adaxial surface and sparsely barbate (*vs.* confluent at apex, glabrous), staminodes obvious, 1–1.3 cm long, sparsely pubescent (*vs.*ca. 1.5 mm long, glabrous). Detailed morphological comparisons with *P.orthandra* are provided in Table 1 and Fig. 2.

**Figure 1.**
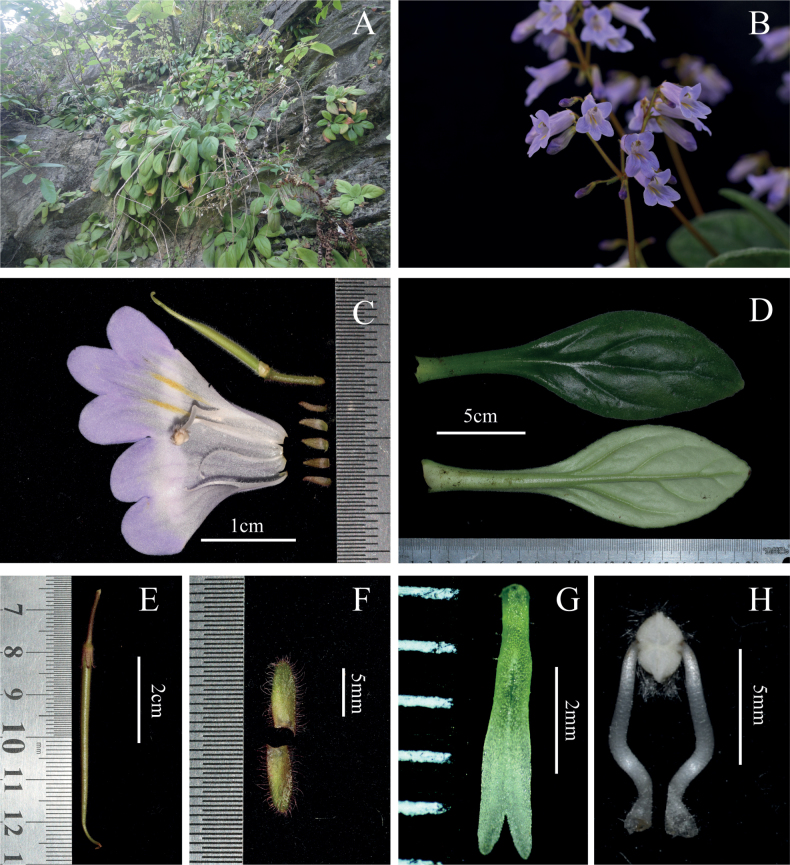
*Primulinapingnanensis* Xin Hong, Z.L.Li & W.C.Chou **A** plants in natural habitat **B** cymes **C** opened corolla and dissected calyx lobes with pistil **D** leaves (up: adaxial surface, down: abaxial surface) **E** young fruit **F** bracts **G** stigmas **H** stamens.

**Figure 2.**
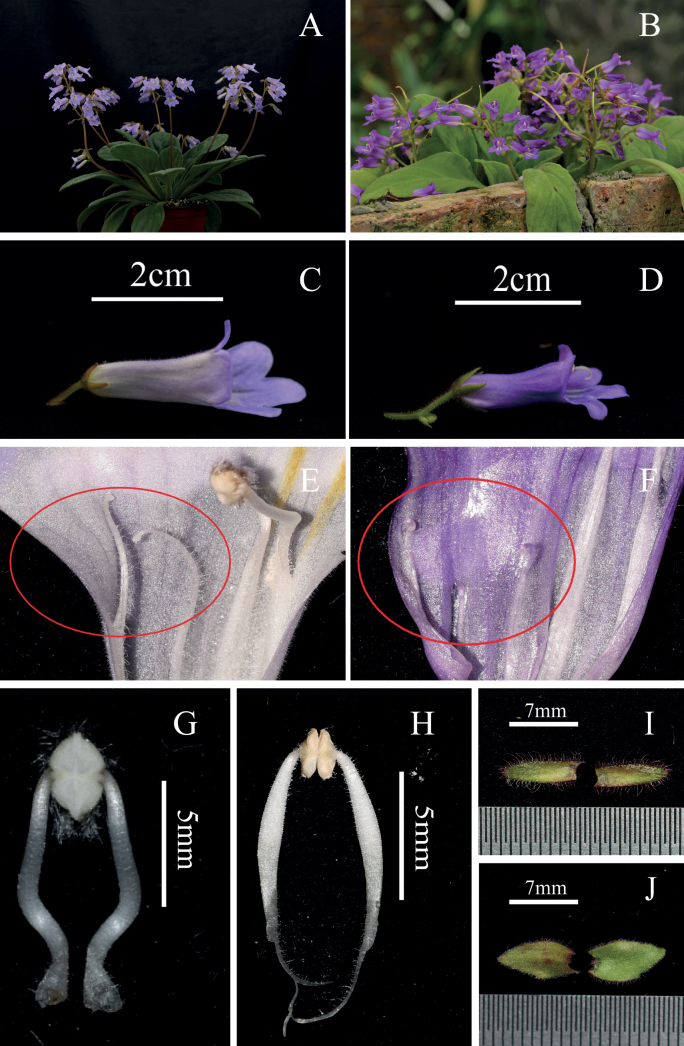
Morphological comparisons between *Primulinapingnanensis* and *P.orthandra***A** habit of *P.pingnanensis***B** habit of *P.orthandra***C** lateral view of mature flower of *P.pingnanensis***D** lateral view of mature flower of *P.orthandra***E** staminodes of *P.pingnanensis***F** staminodes of *P.orthandra***G** filaments of *P.pingnanensis***H** filaments of *P.orthandra***I** bracts of *P.pingnanensis***J** bracts of *P.orthandra*.

**Table 1.** Diagnostic character differences between *Primulinapingnanensis* and *P.orthandra*.

Character	*Primulinapingnanensis*	*P.orthandra*
Bracts	lanceolate	ovate
Tube	funnel-form, no constriction in the middle	tube near tubular, constriction in the middle
Outer corolla surface	sparsely white puberulent	glabrous
Corolla lobes	lobes oblong	lobes broadly ovate
Filaments	strongly bent at the middle	straight
Anthers	fused by the entire adaxial surface and sparsely barbate	confluent at apex, glabrous
Staminodes	obvious, 1–1.3 cm long, sparsely pubescent	ca. 1.5 mm long, glabrous

#### Type.

China. Guangxi Zhuangzu Autonomous Region: Guigang City, Pingnan County, Shuangma Village, 23°37'N, 110°19'E, growing atop a cliff on a limestone hill, 28 March 2018, *Chou Wei-Chuen, CWC180328-01* (holotype: IBK!; isotypes: AHU!, IBK!).

#### Description.

Perennial herb. Rhizomatous stem subterete. ***Leaves*** basal, opposite, and congested at rhizome apex after years of growth; petiole 5–8 cm long, densely pubescent. Leaf blade dark green, thick herbaceous, elliptic to ovate-elliptic, 15–20 × 6–8 cm, apex acute to obtuse, base broadly cuneate, slightly oblique, margin sinuate, densely pubescent on both surfaces; 3–4 pairs of lateral veins on each side of the midrib; adaxially midrib main vein conspicuously sunken and lateral veins inconspicuously sunken, abaxially midrib main vein and lateral vein conspicuously raised. ***Cymes*** 1–7, axillary, 1–3-branched, 1–8(–16)-flowered; peduncles 18–25 cm long, densely brown puberulent and pubescent; bracts 2, opposite, lanceolate, ca. 7 × 2.5 mm, densely brown pubescent on both surfaces. Pedicels 2.5–4 cm long, densely brown puberulent, and pubescent. Calyx 5-lobed from the base, lobes narrowly lanceolate to triangle, apex acute, ca. 4 × 0.9 mm, outside pubescent, inside glabrous. ***Corolla*** zygomorphic, lilac, –4.5 cm long, outside sparsely white puberulent, inside glabrous; tube funnel-form, 2.0–3.0 cm long, 1.0–1.3 cm in diameter at the mouth, ca. 5 mm in diameter at the base; limb distinctly 2-lipped; dorsal lip 2-lobed to the middle, lobes ca. 1.0 cm long, apex suborbicular; ventral lip 3-lobed to the middle, lobes oblong, 1.0–1.5 cm long. ***Stamens*** 2, glabrous, adnate to the corolla tube for ca. 10 mm above the base; filaments 8–10 mm long, extending outwards at the base of the corolla, strongly curved at 90° degrees at the middle, sparsely white pubescent at base; anthers white, ca. 2 mm long, fused by the entire adaxial surface, sparsely barbate; Staminode 3, lateral ones obvious, linear, sparsely pubescent, 1–1.3 cm long, adnate to the corolla for 8 mm above the base; the middle one capitate, ca.1 mm long, adnate at the corolla base. Disc annular, slightly oblique, ca. 1.5 mm in height, margin repand. ***Pistil*** 2.8–3.2 cm long; ovary ca. 1.8 cm long, densely white pubescent; style ca. 1 cm long, densely puberulent; stigma trapeziform, ca. 2 mm long, apex slightly 2-lobed, lobes obtuse triangle, ca. 0.4 mm long. ***Capsule*** 4.5–6 cm long, sparsely brown puberulent, narrowly oblong-ovoid, dehiscing loculicidally into two valves.

#### Phenology.

Flowering from the second half of March to the first half of April, fruiting from June to July.

#### Etymology.

The specific epithet is derived from the type locality, Pingnan County. This county is the birthplace of Mr. Chou’s mother. Thus, Mr. Chou strongly suggested using “*pingnanensis*” as the scientific name.

#### Vernacular name.

Píng Nán Bào Chūn Jù Tái (Chinese pronunciation); 平南报春苣苔 (Chinese name).

#### Distribution and habitat.

*Primulinapingnanensis* is only known from the type locality, Shuangma Village, Pingnan County, Guigang City, Guangxi Zhuangzu Autonomous Region, growing on limestone cliffs at an elevation of ca. 50 m. The average temperature in the distribution area is 22.6 °C, while the average annual precipitation is 1050–2100 mm. The habitat is very close to the village with human activities.

#### Preliminary conservation assessment.

The terrain in the North and South of Pingnan County is mountainous, while the central area is flat. Very few limestone hills are in the plains, but some mountains are near Shuangma Village. After finding the new species, we conducted several detailed explorations of the area. The current survey results showed that this species has a small population at the top of a limestone mountain near Shuangma Village, with fewer than 200 individuals. According to the results of our field investigations in the type locality and adjacent regions, the EOO and AOO of *Primulina*pingnanensis are about 2 km^2^ and 0.1 km^2^, respectively. The severe drought in the second half of 2022 has seriously affected the population. According to the field survey, it is preliminarily estimated that the number of individuals in this population has decreased by 40% or more. More in-depth habitat surveys are warranted to determine if there are more populations nearby. For this current study, we temporarily assess the status of this species as Critically Endangered [CR B1+B2ab (iii, v)], according to the IUCN Red List Categories and Criteria (IUCN 2022).

## ﻿Notes

### ﻿Validation of new combination in *Primulina*

When the new combination for *Primulinacrassifolia* was made by Anh et al. (2022), it was later noticed that the combination was invalid. Therefore, we validate it here.

#### ﻿ Primulina crassifolia

Taxon classificationPlantaeLamialesGesneriaceae

(Aver. & K.S.Nguyen) T.T.P.Anh, F. Wen & Mich.Möller comb. nov.

710934C9-2475-5417-948A-093AE44ABEF3

urn:lsid:ipni.org:names:77324169-1

Chirita crassifolia Aver. & K.S.Nguyen, Pl. Diversity Fl. Veg. Bat Dai Son 254 (2021). Basionym.

##### Type.

Vietnam, Ha Giang Province: Quan Ba district, Tung Vai commune, steep rocky slopes to large and deep cave composed of highly eroded marble-like white limestone, elevation 900–1000 m a.s.l., 17 October 2018, L. Averyanov, Nguyen Sinh Khang, T. Maisak, Truong Duc Thieu, VR 938 (holotype: LE!; isotypes: HN!, LE!).

## ﻿Discussion

The Karst and Danxia landforms from south & southwest China to the north Indo-China Peninsula are well-known for their high species diversity and endemism levels. Some interesting genera of Gesneriaceae for example, *Primulina*, *Petrocodon* Hance, and *Hemiboea* Clarke have been attracting the attention of botanists and taxonomists in the past decades. *Primulina* speciation is positively associated with changes in past temperatures and East Asian monsoons. Climatic change around the mid-Miocene triggered an early burst (Kong et al. 2017; Xu et al. 2019; Hsieh et al. 2022). There is abundant diversity in the morphology of the vegetative and reproductive organs of *Primulina*. Still, it can be distinguished by its rosette leaves, tubular corolla, parietal placentation, etc. We concluded that *P.pingnanensis* is an undescribed species of *Primulina* based on extensive investigation in south and southwest China. A comparison of known live plants and specimens of the genus *Primulina*. *P.orthandra* can be regarded as similar to *P.pingnanensis* because they both have similar leaf morphology, calyx, and purple corolla. However, the differing characteristics of the peduncle, bract, stamen, and staminodes can quickly distinguish the two species from each other.

The high species diversity and restricted distributions on limestone habitats have made the calciphilous *Primulina* an ideal study subject for understanding plant radiation on Sino-Vietnamese limestone karsts (Wei et al. 2022). The habitat of *P.pingnanensis* is located in the mountains in the middle of Pingnan County. The rest of the area is a flat plain. The spatial heterogeneity has created a unique species of *Primulina* in Pingnan. This suggests that the karst areas in southern China have acted as both “museums” and “cradles” of plant evolution (Xu et al. 2019). The discovery and publication of this new species supplements the diversity of *Primulina* distributed in Guangxi. It further indicates that there are more undiscovered new species of *Primulina* in the vast karst areas of south China (Hong et al. 2020; Fu et al. 2022).

## Supplementary Material

XML Treatment for Primulina pingnanensis

XML Treatment for Primulina crassifolia

## ﻿Additional information

### Author ORCIDs

Zheng-Long Li https://orcid.org/0009-0009-8988-5725

Yan-Yun Kuang https://orcid.org/0009-0001-4964-3969

Qing-Qing Xu https://orcid.org/0009-0007-8725-9291

Wei-Chuen Chou https://orcid.org/0000-0001-6470-9295

Xin Hong https://orcid.org/0009-0003-9477-2414

Li Ding https://orcid.org/0009-0003-1523-1784

### Data availability

All of the data that support the findings of this study are available in the main text.

## References

[B1] Anh TT Averyanov LV Möller M Maisak TV Nguyen KS Quang BH Do TN Li RF Wen F (2022) One transfer to *Primulina* (Gesneriaceae) and amended descriptions for *P.crassifolia* and *P.quanbaensis* from northern Vietnam. Nordic Journal of Botany 2022(5): e03455. 10.1111/njb.03455

[B2] Averyanov LV Nguyen SK Tran TH Averyanova AL Maisak VT Nguyen HT (2020) Plant Diversity, Flora and Vegetation of Bat Dai Son Mountain Area, Northern Vietnam. Saint-Petersburg, Strata, 561 pp.

[B3] Du C Liu J Ye W Liao S Ge BJ Liu B Ma JS (2021) Annual report of new taxa and new names for Chinese plants in 2020. Shengwu Duoyangxing 29(8): 1011–1020. 10.17520/biods.2021122

[B4] Du C Liu J Ye W Liao S (2022) 2021 annual report on new taxa and nomenclatural changes of Chinese plants. Shengwu Duoyangxing 30(8): 22207. 10.17520/biods.2022207

[B5] Fu LF Monro AK Wei YG (2022) Cataloguing vascular plant diversity of karst caves in China. Shengwu Duoyangxing 30: е21537. 10.17520/biods.2021537

[B6] GRC (2022) Gesneriaceae Resource Centre. Royal Botanic Garden Edinburgh. [continuously updated ; Retrieved/Accessed: 21 November 2022] https://padme.rbge.org.uk/GRC

[B7] Hao Z Kuang YW Kang M (2014) Untangling the influence of phylogeny, soil andclimate on leaf element concentrations in a biodiversity hotspot. Functional Ecology 29(2): 165–176. 10.1111/1365-2435.12344

[B8] Hong X Keene J Shan WY Do VT Wen F (2020) Taxonomic identity of *Primulinaswinglei* (Gesneriaceae). Guihaia 40(10): 1393–1401.

[B9] Hsieh CL Xu WB Chung KF (2022) Plastomes of limestone karst gesneriad genera *Petrocodon* and *Primulina*, and the comparative plastid phylogenomics of Gesneriaceae. Scientific Reports 12(1): е15800. 10.1038/s41598-022-19812-2 PMC9500069 36138079

[B10] IUCN (Standards and petitions committee) (2022) Guidelines for Using the IUCN Red List Categories and Criteria. Version 15. Prepared by the Standards and petitions committee. https://www.iucnredlist.org/resources/redlistguidelines [accessed on 4 Mar. 2022]

[B11] Kong HH Condamine FL Harris AJ Chen JL Pan B Möller M Hoang VS Kang M (2017) Both temperature fluctuations and East Asian monsoons have driven plant diversification in the karst ecosystems from southern China. Molecular Ecology 26(22): 6414–6429. 10.3897/phytokeys.127.35445 28960701

[B12] Li S Xin ZB Chou WC Huang Y Pan B Maciejewski S Wen F (2019) Five new species of the genus *Primulina* (Gesneriaceae) from Limestone Areas of Guangxi Zhuangzu Autonomous Region, China. PhytoKeys 127: 77–91. 10.3897/phytokeys.127.35445 31379451 PMC6661265

[B13] Möller M (2019) Species Discovery in Time: An Example from Gesneriaceae in China. Guangxi Sciences 26(1): 1–16.

[B14] Pan B Wang BM Yang LH Lai BD Li PW (2022) *Primulinafangdingii* (Gesneriaceae), a new species from Guangxi, China. Nordic Journal of Botany 2022(10): e03684. 10.1111/njb.03684

[B15] Wang WT Pan KY Li KY (1990) Gesneriaceae. In: Wang WT (Ed.) Flora Reipublicae Popularis Sinicae (Vol.69). Science Press, Beijing, 141–271.

[B16] Wang WT Pan KY Li ZY Weitzman AL Skog LE (1998) Gesneriaceae. In: Wu ZY Raven PH (Eds) Flora of China (Vol.18). Science Press, Beijing & Missouri Botanical Garden Press, St. Louis, 254–401.

[B17] Wei YG (2018) The Distribution and Conservation Status of Native Plants in Guangxi, China. China Forestry Publishing House, Beijing, 876 pp.

[B18] Wei YG Do VT Wen F (2022) A Checklist to the Plants of Northern Vietnam. China Forestry Publishing House, Beijing, 606 pp.

[B19] Xin ZB Li S Zhang RL Fu LF Dong J Wen F (2018) *Primulinazhoui* and *P.huangii* (Gesneriaceae), two new species from limestone areas in Guangxi, China. Taiwania 63(1): 54–60. 10.6165/tai.2018.63.54

[B20] Xiong C Chou WC Huang Y Wen F (2022) *Primulinanana* (Gesneriaceae), a new species from eastern Guangxi, China. PhytoKeys 197: 33–39. 10.3897/phytokeys.197.83089 36760675 PMC9848987

[B21] Xu M Yang L Kong H Wen F Kang M (2019) Congruent spatial patterns of species richness and phylogenetic diversity in karst flora: Case study of *Primulina* (Gesneriaceae). Journal of Systematics and Evolution 59(2): 251–261. 10.1111/jse.12558

